# Localization-Free Detection of Replica Node Attacks in Wireless Sensor Networks Using Similarity Estimation with Group Deployment Knowledge

**DOI:** 10.3390/s17010160

**Published:** 2017-01-15

**Authors:** Chao Ding, Lijun Yang, Meng Wu

**Affiliations:** 1 College of Computer Science, Nanjing University of Posts and Telecommunications; Nanjing 210003, China; dingchao_129@163.com; 2 College of Internet of Things, Nanjing University of Posts and Telecommunications; Nanjing 210003, China; 3 Key Lab of “Broadband Wireless Communication and Sensor Network Technology” of Ministry of Education, Nanjing University of Posts and Telecommunications; Nanjing 210003, China

**Keywords:** security in wireless sensor networks, replica node detection, location similarity, deployment knowledge

## Abstract

Due to the unattended nature and poor security guarantee of the wireless sensor networks (WSNs), adversaries can easily make replicas of compromised nodes, and place them throughout the network to launch various types of attacks. Such an attack is dangerous because it enables the adversaries to control large numbers of nodes and extend the damage of attacks to most of the network with quite limited cost. To stop the node replica attack, we propose a location similarity-based detection scheme using deployment knowledge. Compared with prior solutions, our scheme provides extra functionalities that prevent replicas from generating false location claims without deploying resource-consuming localization techniques on the resource-constraint sensor nodes. We evaluate the security performance of our proposal under different attack strategies through heuristic analysis, and show that our scheme achieves secure and robust replica detection by increasing the cost of node replication. Additionally, we evaluate the impact of network environment on the proposed scheme through theoretic analysis and simulation experiments, and indicate that our scheme achieves effectiveness and efficiency with substantially lower communication, computational, and storage overhead than prior works under different situations and attack strategies.

## 1. Introduction

Low-power wireless sensor networks (WSNs) are known to be capable of rapid deployment in large geographical area in a self-organized manner, which makes them particularly suitable for real-time large-scale data collection and event monitoring for mission-critical applications, such as border monitoring, target tracing, and in-network aggregation. In such applications, the sensors are deployed in a hostile environment with potential security threats. However due to the constraints of network scale and fabrication cost, the sensor nodes are usually exploited by adversaries with poor security guarantees. Meanwhile, since in most cases WSNs are remotely administrated by the network operator, the sensor nodes are often deployed in an unattended manner. Thus, compared with traditional wired and wireless networks, WSNs are much more vulnerable to a variety of attacks from the inside and outside of the network, such as eavesdrop, forge, and node compromise. In recent years a large amount of research efforts [1,2,3,4,5] focus on the security issues of WSNs and their corresponding fields.

Among such attacks, the node replica attacks [6] may be particularly dangerous to WSNs because it is difficult for the security mechanisms to identify the replica nodes with a reasonable time and resource consumption. However, once an adversary possesses a small number of compromised nodes, he can easily generate a large number of replicas which share the keying materials and IDs with the original ones, spreading the replicas throughout the network. The sensor nodes, which pass the verification of the network security protocols, are able to create pairwise shared keys with other nodes and the basestation (BS) with legal keying materials and IDs and, thus, capable of encrypting, decrypting, and authenticating their own communications on demand, as if they were the original compromised ones. To our best knowledge, the cost of generating replicas for the adversary is much lower than that of compromising equal quantities of sensor nodes, which makes it extremely economical to launch node replica attacks. By injecting a large number of replicas into the target network, the adversary manages to continuously undermine the network without being detected. For example, he can overhear the traffic pass through his deployed replicas, and inject false data to disturb the data collection. Alternatively, he could adopt more aggressive strategies which undermine the network protocols such as clustering and in-network aggregation, thereby incurs continuous harm to the network operations. To some extents, node replica attack are far more dangerous than node compromise attacks, as the time and effort spent on the node replication are much less. However, compared with other security threats, like eavesdropping, forgery, denial of service, and node compromise, the node replica attack receives much less attention. We, thus, believe that it is necessary to develop distributed lightweight countermeasures to address the threat of node replicas in an early stage of network.

A straightforward solution to the node replica problem is to equip the tamper-proof hardware on each node in the network against illegal loading of security materials and malicious program rewriting. However such a solution is much too expensive for most sensor network applications. Additionally, although tamper-proof hardware has the adversaries spending more time and effort on node compromise, it may still be possible to bypass tamper-resistance for a small number of nodes in reasonable amounts of time. Another class of solutions [6,7,8,9,10,11] identifies the replica nodes based on the location claims reported by the sensor nodes themselves. These solutions deduce the location anomalies based on the conflicts existing in the location claims. However, these location-claim-based schemes are vulnerable to the falsified location claims generated by the replicas. The replica nodes manage to elude the detection by reporting the same location as the original compromised nodes to BS.

To address the limitation of the prior works, we propose a location-free scheme to detect node replica attacks in sensor networks using group deployment knowledge. Our scheme adapts the location claim idea presented in [6]. The basic idea behind our proposal is that it is reasonable to treat a node as a replica when its claimed location is far away from its true location. However, the exact physical positions of sensor nodes are difficult to obtain since accurate localization is not practical in sensor networks due to high cost and various types of environmental uncertainties. Thus, instead of deploying resource-consuming localization techniques on resource-constrained sensor nodes, we design a novel metric named location similarity to quantify the deviation between true and claimed location using locality sensitive hashing (LSH) based similarity estimation techniques [12]. Such a metric only requires the sensor nodes to collect their neighbors’ IDs as well as receiver signal strength indicator (RSSI) [13] of the top four nearest deployment points. In addition, our scheme works on the basis of the assumption that sensor nodes are deployed in groups and the nodes in each group are placed around the predefined location named deployment point. In such a case, our work allows most nodes within a group to communicate without generating any location claims. Due to the aforementioned advantages, our scheme achieves an effective and efficient replica detection with low communication, computation, and storage overhead.

Furthermore, we validate the security performance of the proposed scheme through heuristic analysis under different attack strategies and demonstrate that our scheme provides robust replica detection even under the condition that there are considerable nodes compromised by adversaries. In addition, we also evaluate the effectiveness and efficiency of the proposed scheme through both theoretic analysis and simulation experiments. The results show that our approach achieves effective and efficient replica detection while incurring significantly low overhead.

The rest of paper is organized as follows: in Section 2, we propose the preliminaries of this paper, including the network assumptions, attacker models and the group based random deployment strategy. In Section 3, we present the mathematical definition of the node replica attack and the location similarity, In Section 4, we describe the details of the proposed LR2ND scheme. In Section 5 and Section 6, we present the security analysis and the performance evaluation, respectively. Finally in Section 7, we summarize our work.

## 2. Related Works

The discussion about the replica node attack in WSNs was firstly found in Parrno et al’s. work [6], in which randomized multicast and line-selected multicast schemes are proposed to address such problems. In the Randomized Multicast scheme, signed location claims are sent to randomly chosen witness nodes for the validation of consistency. A node will be considered to be replicated if two conflicting location claims about this node are found. The improved line-selected scheme effectively reduces the communication overhead incurred by location claim transmission of the randomized multicast scheme by having every claim-relaying node participate in the replica detection and revocation process. However, these multicast-based schemes [6] and their variants [7], have to periodically multicast the location claims over the whole lifetime of the network, resulting in very large communication and computation overhead. In our scheme, replica detection works on the basis of group deployment knowledge. Only the nodes placed outside its home group are required to send location claims, which achieves significant higher resource efficiency than [6] does.

Based on the line-selected multicast scheme of [6], Conti et al. [8] proposed a randomized improved scheme RED to enhance the performance in terms of replica probability, storage and computation overheads. However, compared with [6], the communication resource efficiency of RED scheme has no significant improvement. Furthermore, the protocols require repeated claims over time, which means that the communication overhead of such scheme needs to be multiplied by the number of runs during the entire network lifetime. In contrast, our proposed scheme achieves higher communication resource efficiency than RED by only requiring location claims when new arrivals are placed in the network.

Abinaya et al. [9] proposed the improved scheme X-RED on the basis of RED [8]. The main design principle of X-RED is similar to RED, but the witness is selected dynamically using a randomized hash function. The approach of randomized witness selection can evenly distribute overhead among nodes, which effectively prevent single point of failure. However, the drawback of very large overhead caused by periodic claim examination is still not improved in X-RED.

Zhu et al. [7] proposed a replica detection scheme based on grid cell topology, which detects replicas by multicasting location claim to single cell or multiple cells. The chief advantage is that it enhances the detection accuracy of schemes proposed in [6]. However, its communication overhead has no significant improvement compared with [8]. Our scheme can achieve similar detection accuracy with much lower communication overhead.

Choi et al. [10] proposed a localized replica detection scheme for sensor networks based on regionalized deployments. In this work, the network is viewed as a subsets of non-overlapping subregions, each of which has an exclusive subset. If the intersection of these subsets is not empty, it is reasonable to imply that replicas are included in such subsets. However, the adversary can bypass the detection of Choi’s et al. work by some specific replica placing methods. Our scheme can effectively address this problem.

Ho et al. [11] proposed replica detection schemes based on group deployment knowledge which adapts the location claim idea from [6]. In these schemes, the sensor nodes inside their home zone can transmit their ordinary messages without any extra validation of security protocols, whereas the sensor nodes outside their home zone are not allowed to transmit messages unless they are authenticated by location claims. Compared with prior works, this scheme eliminates most of the communication, computational, and storage overheads, since it only requires part of nodes to generate and send their location claims. However, the scheme is built on the assumption that every node knows its own position by some kind of localization protocols, and its detection performance depends on the accuracy of localization. Actually, it is very expensive to deploy localization schemes on the resource-limited sensor nodes, and accurate localization is hard to get since there are various uncertainties in WSNs. In addition, the usage of localization may introduce more potential threats due to the security vulnerabilities of existing localization schemes [14]. Our scheme achieves secure, effective, efficient replica detection with similar low communication, computational, and storage overheads in a localization-free manner.

Khedim et al. [15] propose a mobile assistant clone detection (MCD) protocol aiming at mitigating the dependence on the GPS and beacon nodes. MCD is a hybrid protocol which uses patrol robots and honeypots for the node replication detection in static sensor networks to enhance the detection performance. However this scheme requires extra expensive hardware which substantially increases the deployment costs. Meanwhile the scheme requires periodic examination on all of the nodes in the network over the entire network lifetime. In contrast, the proposed scheme only starts the node replication detection on demand and the detection is limited in a local region. Chen et al. [16] propose an intrusion detection algorithm to address the problem of replication attacks in the clustered wireless sensor networks based on a novel clustering protocol NI-LEACH. The main advantage is that the scheme is configurable according to the performance requirements by choosing appropriate encoder functions. However, this scheme requires the witness nodes to be randomly selected from network in order to undertake large amount of computation intensive and energy consuming tasks. The sensor nodes which act as witnesses will rapidly run out of energy.

Ho et al. [17] propose a node replication detection scheme which is composed of quorum-based multicast (QBM) and star-shape line-selected multicast (SLSM), which can deterministically detect the replicas. However this scheme still requires repeated claim checking, which results in large amount of communication.

Additionally, a Sybil attack [18] can be regarded as an extended form of node replication attacks, there are also some typical schemes. Pecori [19] proposes a security protocol which resists Sybil attacks through the use of a combined trust-based algorithm exploiting reputation techniques. Compared to similar methods, such a trust-based algorithm shows promising results in thwarting a Sybil attack in a Kademlia network.

## 3. Preliminaries

In this section, we first present the underlying assumptions and sensor deployment strategy, and then describe the detailed attack model of our scheme.

### 3.1. Network Assumptions

We assume that the proposed scheme works in a typical two-dimension static sensor networks in which every node holds their own position immediately after deployment. All direct communications links in the network are bidirectional. This assumption is common in the current generation of sensor networks. We assume that the sensor nodes in the network can be divided into two categories from the duty perceptive: the ordinary nodes and BS, where the ordinary nodes generate ordinary data and location claim related messages, and send them to BS via single- or multi-hop transmission, whereas BS collects location claims for further analysis of similarity estimation and the final decision on the suspicious replicas. We also assume that the sensor network are deployed in an open space which allows us to perform distance measurement using the Receiver signal strength indication (RSSI) extracted from media access control layer protocols. This assumption is common in current generation of WSNs. Additionally, BS may take further security measurements such as software attestation and node revocation if necessary. We also assume that BS is a trusted entity. This is a common and reasonable assumption since if BS is compromised, the sensor networks suffers a risk of a single-point of failure, which means that the entire mission of the sensor network can be easily undermined.

Furthermore, we assume that each node in the network has and only has a unique ID so that BS is able to correctly parse the source of location claims. Moreover, we assume that the message authentication code (MAC) is adopted to filter the unauthorized modification on the network traffic and verify the message source. In this work we adopt the MAC algorithm proposed in [20], whose main advantage is that the authentication tag of such a MAC algorithm can be aggregated, resulting in a substantial reduction of the location claim size.

### 3.2. Sensor Deployment Strategies

**Figure 1:**
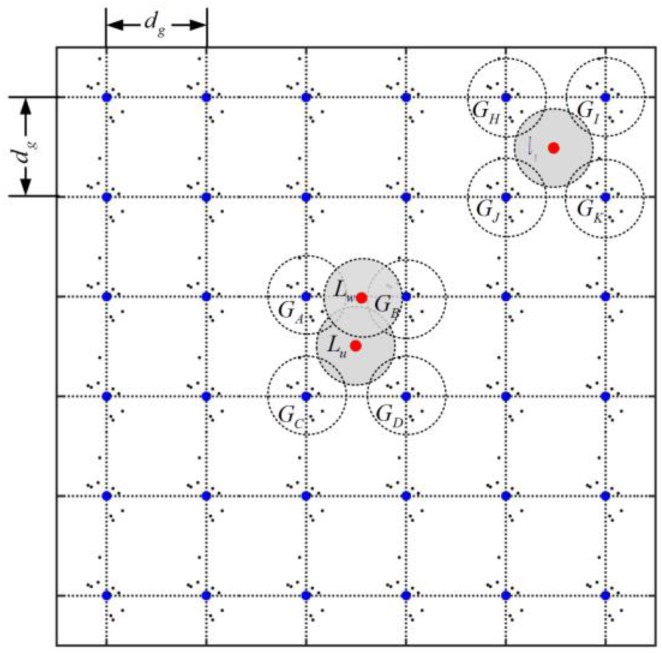
The group-based random deployment strategy for WSNs.

We adopt a group-based random deployment strategy in our scheme. In this work, we assume that the whole network is divided into grids as shown in Figure 1, and define the grid intersection points as predefined deployment points. Before deployment, we firstly place sensor nodes exactly at these deployment points as beacons. The rest of sensor nodes are allocated into groups and programed with the corresponding group information, such as Group ID. We assume that the number of nodes in each group is even. Then, during the deployment, the nodes in the same group are randomly placed around corresponding deployment point. We assume that the coordination of the sensor nodes within one single group follows the two-dimensional Gaussian distribution. This is reasonable and practical deployment strategy since in most sensor network applications sensor nodes are spread over the target region in a randomly scatter manner such as dropped from airplane or spread by hand. This assumption is supported by the fact that the group deployment strategy has been used for various applications in sensor networks, such as key distribution [21,22] and public key authentication [23].

**Figure 2:**
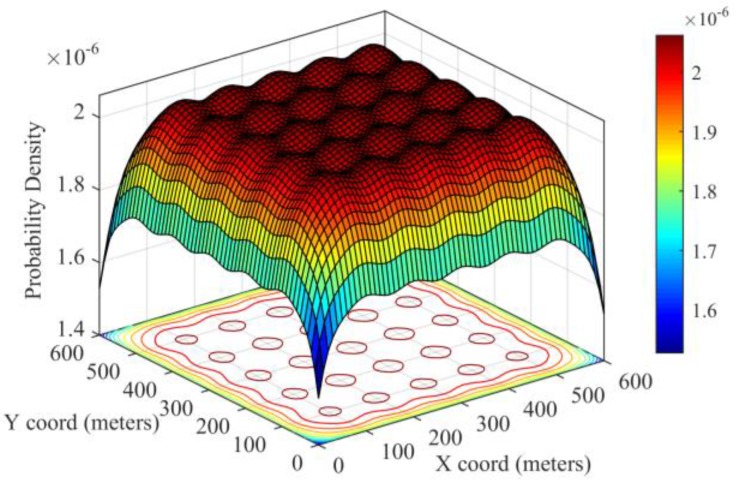
The overall probability distribution over the entire deployment region.

The detailed deployment rules and basic assumptions are described in the form as follows: (1) assuming that there are *m* predefined deployment points that are placed at the grid intersection points, denoted by $g_{1},\;g_{2},\;\ldots ,\;g_{m}$; (2) assuming that there are a total of *M* nodes in the network and these nodes are divided into *m* groups, denoted by $G_{1},\;G_{2},\ldots ,G_{m}$, namely M/m nodes in each group; and (3) the nodes within the same group are *i.i.d.*, following a two-dimensional Gaussian joint distribution. For example, for the node *k* (k=1, 2,…, M/m) in group $G_{i}$ (i=1, 2,…,m), the probability density function of node *k*’s coordination $\left(x_{k},\;y_{k}\right)$ is represented as:

(1) $$f\left(x,\;y|k\in G_{i}\right)=\frac{1}{2\pi \sigma^{2}}\exp\left(-\frac{{\left(x-x_{i}\right)}^{2}+{\left(y-y_{i}\right)}^{2}}{2\sigma^{2}}\right),$$

where $\left(x_{i},\;y_{i}\right)$ is the coordination of predefined deployment point $g_{i}$, whereas the coordination of the sensor nodes from different groups are independent from each other. Figure 2 illustrates the distribution of the probability density distribution over the entire deployment region.

Furthermore, let $l=\sqrt{{\left(x-x_{i}\right)}^{2}+{\left(y-y_{i}\right)}^{2}}$ be the Euclidian distance between node *k* and the predefined position $g_{i}$. Then (1) can be rewritten as:

(2) $$f_{eu}\left(l|k\in G_{i}\right)=\frac{1}{2\pi \sigma^{2}}\exp\left(-\frac{l^{2}}{2\sigma^{2}}\right).$$

### 3.3. Attack Model

In this work, we assume that the adversary can launch a node replication attack by compromising a subset of nodes, generating a large amount of their replicas, and spreading the replicas throughout the networks. Upon compromising a node *u*, the adversary is able to produce a group of replicas $u^{\prime }=\left\{{u^{\prime }}_{1},\;{u^{\prime }}_{2},\;\ldots ,{u^{\prime }}_{r}\right\}$ of which the IDs and secret materials are the same as the original compromised node *u*. The replicas can easily bypass the authenticity and integrity validation of the existing cryptographic security mechanism since they can sign, encrypt, and decrypt the message to play the role just like their original compromised node. Once the replicas are recognized as a legal part of the network, they can launch a variety of attacks, such as false data injection, protocol disruption, and traffic jamming. Moreover, replicas can also assist the original compromised nodes to extend the attack range, as well as reduce the attack cost.

However, we impose several constraints on the adversary’s behaviors. We assume that the adversary is not able to generate new legal IDs since all of the nodes’ IDs are determined before deployment following the deployment strategy. Additionally, the adversary also cannot extract the data in the nodes’ memory before they are compromised. We also assume that the adversary can only compromise a minority of sensor nodes since if he can compromise a major fraction of the network, he will not benefit much from the node replica attack. Furthermore, the adversary would make every effort to extend the deployment range of the replicas; in other words, the replica should be placed at a distance from its origins. Although the replica nodes are hard to detect when they are place close to their original compromised nodes, this will not bring any benefits to the adversary.

The adversary can undermine the location claim-based protocols by deploying large amounts of compromised nodes to report fake locations and participate in local control protocols. However, such an attack strategy requires the adversary to place one compromised node to accompany each replica in the network, resulting in a very high cost for launching node replica attacks. We suppose that the adversary does not adopt this attack strategy. This assumption goes unstated but is implied by the use of signed location claims in other replica detection schemes [6,8]. In addition, it is worthwhile to note that deploying multiple replicas of a single compromised node into the same region does not bring the adversary more benefits. This is because the output of multiple replicas would be treated as redundant and discarded. Multiple replicas with the same ID would not have more influence in a region than a single replica. Furthermore, due to page limitation, in this work we only discuss the case in which no collisions exist between the compromised nodes and their replicas.

## 4. Localization-Free Replica Detection Based on Similarity Estimation

In this section, we first present a formal statement of the node replica problem, and propose a neighborhood relationship knowledge-based metric that allows us to detect replicas without assistance of nodes’ positions using local sensitivity hashing (LSH)-based location similarity estimation techniques. We then describe the details of proposed protocol that stop node replica attacks without resource-consuming localization mechanisms. Finally we present a simulation model that estimate the detection threshold of the proposed scheme. Table 1 lists the most frequently used notation in this paper.

### 4.1. Problem Statement

Once the adversary succeeds in compromising a node, he can create replica nodes as follows: he extracts ID and all secret materials from the compromised nodes and loads these key information into the replicas so that the replica has the same ID and secret materials as its origin node. The adversary can compromise multiple nodes and generate multiple replicas of a single compromised node. For a specific compromised node *v*, there may be *n* replica nodes in the network, denoted by $v_{1},\;v_{2},\ldots ,v_{n}$. We assume that compromised node *v* is placed at the location $L_{v}\;\left(x_{v},\;y_{v}\right)$ while the replicas are placed at locations $L_{v_{k}}\left(x_{v_{k}},\;y_{v_{k}}\right)$, k=1, 2, …,n. To bypass the location claim conflict detection, the replicas may falsify the location claims denoted by ${L^{\prime }}_{v_{k}}\left({x^{\prime }}_{v_{k}},{y^{\prime }}_{v_{k}}\right)$, which equals to $L_{v}$. A straightforward solution for this problem is to build a threshold-based detection mechanism on the distance between the claimed location ${L^{\prime }}_{v_{k}}$ and the true location $L_{v_{k}}$. However, the true location $L_{v_{k}}$ is not visible for BS without accurate localization by multiple witness nodes. Actually, in most sensor network applications, accurate localization is difficult to achieve due to strict resource constraints. The inaccuracy existing in localization may incur large amounts of uncertainties and false alarms in the replica detection schemes. How to handle these uncertainties and enhance the effectiveness with a substantially low communication, computation, and storage overheads is an important issue explored in this paper.

### 4.2. Neighborhood-Based Detection Metric

To address the limitation of the localization based replica detection schemes, we propose a novel detection metric named location similarity based on neighborhood relationship knowledge. The basic idea behind location similarity is that the neighborhood situation of two nodes should be very different when they are far away from each other. Taken the deployment situation shown in Figure 1 as example, the nodes *u*, *v*, and *w* are placed at locations $L_{u}$, $L_{v}$, and $L_{w}$, respectively. The location $L_{u}$ has quite a long distance from $L_{v}$, while $L_{u}$ are near $L_{v}$. From Figure 1, we can see that the neighboring nodes of *u* mostly reside in the groups $G_{A},\;G_{B},\;G_{C}$, and $G_{D}$, which is quite different from the neighboring nodes’ distribution of node *v* (in $G_{H},\;G_{I},\;G_{J},\;G_{K}$), but similar to the neighboring distribution of node *w*. Hence, we believe that it is reasonable to judge a node as a replica as long as the deviation of the neighboring node distribution between its claimed location and true location exceeds a predefined threshold.

We first introduce a notation *neighboring vector* to indicate the distribution of one node’s neighborhood. We further define *derived neighboring vector* as the vector that indicates the neighboring distribution derived from one node’s claimed location. Then we have:

**Definition 1** (Neighboring Vector (NV))**.** *For any node u, the normalized vector* $S_{nei}\left(u\right)$ *is NV if and only if*

(3) $$S_{nei}\left(u\right)=\frac{\left(S_{L_{u}}^{\left(G_{1}\right)},\;S_{L_{u}}^{\left(G_{2}\right)},\;\ldots ,S_{L_{u}}^{\left(G_{m}\right)}\right)}{\sum_{i=1}^{m}S_{L_{u}}^{\left(G_{i}\right)}},$$

*where m is the number of groups while* $S_{L_{u}}^{\left(G_{i}\right)}$ *accounts for the number of u’s neighboring nodes which belongs to group* $G_{i}$ *when u is at its true location* $L_{u}$*.*

According to Definition 1, we try to find out the mathematical mapping relationship between NV and the physical location based on the group deployment knowledge so that we can derive the neighboring distribution from the claimed location of sensor nodes. Theorem 1 and Inference 1 show such a relationship.

**Theorem** **1.** *Let* $R_{z}$ *be the communication radius of a sensor node. Let* $g\left(z|k\in G_{i}\right)$ *be the probability that node k from group* $G_{i}$ *resides within the neighborhood of a node which is z distance from the predefined deployment point* $g_{i}$*. Then the probability* $g\left(z|k\in G_{i}\right)$ *is:*

(4) $$g\left(z|k\in G_{i}\right)=I\left\{z<R_{z}\right\}\left[1-e^{-\frac{{\left(R_{z}-z\right)}^{2}}{2\sigma^{2}}}\right]+\int_{\left|z-R_{z}\right|}^{z+R_{z}}f_{eu}\left(l|k\in G_{i}\right)\cdot 2l\;\arccos\left(\frac{l^{2}+z^{2}-{R_{z}}^{2}}{2lz}\right)dl,$$

*where function* $f_{eu}\left(l|n_{i}\in G_{i}\right)$ *is the node distribution function illustrated in Equation (2), constant value* $R_{z}$ *represents the communication radius of each node, and* I{⋅} *is the set indicator function. The value of* I{⋅} *is 1 when* $z<R_{z}$ *holds, and 0 otherwise.*

**Figure 3:**
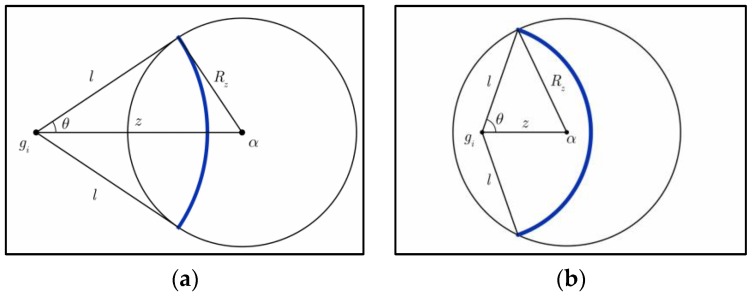
Probability of the nodes which are *l*-distance from $g_{i}$ residing in α’s communication range: (**a**) $z\geq R_{z}$; (**b**) $z<R_{z}$.

**Proof.**  For group $G_{i}$, the sensor nodes that are *l-*distance from deployment point $g_{i}$ should reside in a circle which is centered at $g_{i}$ with the radius l. If these nodes also reside in the communication range of the node *u*, they should reside in a circle which is centered at *u* with the radius $R_{z}$. In other words, as illustrated in Figure 3a,b, the nodes that satisfy the conditions under the lemma hypothesis should resides on the $g_{i}$’s arc within the *u*’s circle. Let $L_{arc}\left(l,\;z,\;R_{z}\right)$ denote the length of such arc. Based on the node distribution presented in Section 3.2, the probability that the nodes reside in *u*’s communication range when they are *l*-distance from $g_{i}$, denoted by $g\left(z|\mathrm{dist}\left(k,g_{i}\right)=l\right)$, can be derived as the probability that the node *k* falls on an infinitesimal ring area (the bold area in Figure 3) $L_{arc}\left(l,\;z,\;R_{z}\right)\cdot dl$. Then we have:

(5) $$g\left(z|\mathrm{dist}\left(k,g_{i}\right)=l\right)=f_{eu}\left(l|k\in G_{i}\right)\cdot L_{arc}\left(l,\;z,\;R_{z}\right)\cdot dl.$$

Based on the basic geometry knowledge, we can derive the length of arc $L_{arc}$ as:

(6) $$L_{arc}\left(l,\;z,\;R_{z}\right)=2l\cdot \arccos\left(\frac{l^{2}+z^{2}-R_{z}^{2}}{2zl}\right).$$

When the condition $z\geq R_{z}$ holds, the line segments $l,\;z,\;R_{z}$ form a triangle in which *z* is longer than $R_{z}$. Using the triangle axiom, *l* ranges from $z-R_{z}$ to $z+R_{z}$. Then $g\left(z|k\in G_{i}\right)$ can be derived as:

(7) $$\begin{array}{cc} g\left(z|k\in G_{i}\right) & =\int_{z-R_{z}}^{z+R_{z}}f_{eu}\left(l|k\in G_{i}\right)\cdot L_{arc}\left(l,\;z,\;R_{z}\right)\cdot dl\\ & =\int_{z-R_{z}}^{z+R_{z}}f_{eu}\left(l|k\in G_{i}\right)\cdot 2l\cdot \arccos\left(\frac{l^{2}+z^{2}-R_{z}^{2}}{2zl}\right)\cdot dl. \end{array}$$

On the other hand, when the condition $z<R_{z}$ holds, we consider two different cases. In the first case, *l* ranges from $R_{z}-z$ to $R_{z}+z$, the value of $L_{arc}$ is the same as Equation (6). In the second case, *l* ranges from 0 to $R_{z}-z$, the whole circle centered at $g_{i}$ with the radius l resides inside the circle centered at α with the radius $R_{z}$. Then we have:

(8) $$g\left(z|k\in G_{i}\right)=\int_{0}^{R_{z}-z}f_{eu}\left(l|k\in G_{i}\right)\cdot 2\pi l\cdot dl+\int_{R_{z}-z}^{R_{z}+z}f_{eu}\left(l|k\in G_{i}\right)\cdot 2l\cdot \arccos\left(\frac{l^{2}+z^{2}-R_{z}^{2}}{2zl}\right)\cdot dl$$

We can then merge Equations (7) and (8) with the assistant of *indicator function*, as follows:

(9) $$g\left(z|k\in G_{i}\right)=I\left\{z<R_{z}\right\}\left[1-e^{-\frac{{\left(R_{z}-z\right)}^{2}}{2\sigma^{2}}}\right]+\int_{\left|z-R_{z}\right|}^{z+R_{z}}f_{eu}\left(l|k\in G_{i}\right)\cdot 2l\cdot \arccos\left(\frac{l^{2}+z^{2}-{R_{z}}^{2}}{2lz}\right)\cdot dl.$$

Hence, Lemma 1 has been proved. Similar derivation can also be found in [24]. □

Theorem 1 indicates that for a specific node *u*, we can obtain the probability that the nodes from any group $G_{i}$ reside in its neighborhood given node *u*’s radio radius $R_{z}$, and its distance *z* from the corresponding deployment point $g_{i}$. Then we can derive the average number of nodes that reside in node *u*’s communication range for every group in the deployment region given the physical location of *u*. We use a derived neighboring vector (DNV) to describe such average neighboring distribution of a node. Inference 1 shows how we get DNV, based on Theorem 1.

**Inference** **1.** *Given the location* $L_{u}$ *of node u and deployment points* $\left\{g_{1},\;g_{2},\;\ldots g_{m}\right\}$ *over the entire network, the normalized DNV derived by location* $L_{u}$ *can be obtained as*

(10) $$S_{nei-D}=\frac{\left(\mu_{L_{u}}^{\left(G_{1}\right)},\;\mu_{L_{u}}^{\left(G_{2}\right)},\ldots ,\mu_{L_{u}}^{\left(G_{m}\right)}\right)}{\sum_{i=1}^{m}\mu_{L_{u}}^{\left(G_{i}\right)}},\;\mu_{L_{u}}^{\left(G_{i}\right)}=\frac{M}{m}g\left(\mathrm{dist}\left(L_{u},L_{g_{i}}\right)|k\in G_{i}\right),$$

*where M is the total number of nodes in the network, m is the number of deployment groups, whereas* $g\left(\mathrm{dist}\left(L_{u},L_{g_{i}}\right)|k\in G_{i}\right)$ *accounts for the probability that the nodes from group* $G_{i}$ *reside in the communication range of node u.*

**Proof.**  Consider group $G_{i}$, according to the deployment strategy described in Section 3.2, the number of nodes in such group is M/m. Since every node in group $G_{i}$ resides in the communication range of *u* with a success probability $g\left(\mathrm{dist}\left(L_{u},L_{g_{i}}\right)|k\in G_{i}\right)$. The number of nodes from $G_{i}$ residing in *u*’s range is a random value which follows Bernoulli distribution. Hence, the mean of such random values comes to $\left(M/m\right)g\left(\mathrm{dist}\left(L_{u},L_{g_{i}}\right)|k\in G_{i}\right)$. □

Based on the bijective relationship between the physical location and neighboring distribution shown in Theorem 1 and Inference 1, we use the deviation of the neighboring vector instead of the Euclidian distance to characterize the difference between two physical locations. The former is much easier to obtain and compute in a practical sensor network deployment. To further reduce the complexity of computation of inter-vector distance, we adopt the locality sensitive hashing (LSH) coding algorithms, such as MinHash and SimHash [12], to encode the neighboring vector, so that the computation of the inter-vector distance can be simplified as a computation of the Hamming distance between two binary sequence. We define the NV-based location similarity as follows.

**Definition** **2.** *Neighboring vector-based location similarity (NV-LS). Let* $S_{nei-I},\;S_{nei-\mathrm{II}}\in {\mathbb{R}}^{m}$ *denote neighboring vectors corresponding to two different locations. Let* LSH(⋅) *be LSH encoding function whose output is b-bit binary code. According to LSH sequence similarity defined in* [12]*, the NV-LS of* $S_{nei-I}$ *and* $S_{nei-II}$ *is*

(11) $$\mathrm{sim}\left(S_{nei-I},\;S_{nei-II}\right)=1-\frac{D_{h}\left(\mathrm{LSH}\left(S_{nei-I}\right),\;LSH\left(S_{nei-II}\right)\right)}{b},$$

*where* $D_{h}\left(\mathrm{LSH}\left(S_{nei-I}\right),\;LSH\left(S_{nei-II}\right)\right)$ *is the Hamming distance between the binary sequences* $\mathrm{LSH}\left(S_{nei-I}\right),$ $LSH\left(S_{nei-II}\right)$*. The location similarity is a real-value that ranges from 0 to 1. The two locations are very close to each other when the value of location similarity tends to 1.*

### 4.3. Protocol Description

We assume that *M* sensor nodes in the network are divided into *m* groups and each group has M/m nodes. As illustrated in Figure 1, the beacon nodes are placed accurately at the deployment points which are distributed in grids with grid spacing $d_{g}$. Every node has a unique identity (ID) which includes two parts: the node ID (NID) and group ID (GID). We also assume that key materials are pre-loaded to sensor nodes for pairwise key establishment and other security mechanisms. In addition, we adopt an aggregated MAC approach proposed in [20] for all of the MAC generation and verification in our work. The aggregated MAC is a lightweight data integrity verification technique. In our previous works [25], we demonstrate that the aggregated MAC is affordable for sensor networks by evaluating the computation overhead on the MICA2 motes. Additionally, the trust-based scheme [19] is also an alternative solution for the integrity verification. In this approach, the authentication tags can be aggregated by performing an XOR operation, namely the aggregated tag can be obtained by:

(12) $$\mathrm{Tag}=\overset{n}{\underset{i=1}{⊕}}tag_{i}=\overset{n}{\underset{i=1}{⊕}}{\mathrm{MAC}}_{k_{i}}\left({\mathrm{data}}_{i}\right),\;tag_{i}={\mathrm{MAC}}_{k_{i}}\left({\mathrm{data}}_{i}\right).$$

The size of the aggregated tag equals to any single ${\mathrm{tag}}_{i}$. Whereas it can be used to verify the integrity of ${\mathrm{data}}_{1}\|{\mathrm{data}}_{2}\|\ldots \|{\mathrm{data}}_{n}$. The proposed protocol includes three phases as follows:

**Phase 1: Initiation.** We first define a system parameter *trust threshold*, denoted by $d_{\mathrm{th}}$. Consider a particular node *u* from group $G_{i}$; we define $G_{i}$ as *u*’s home group. Node *u* accepts and forwards the messages from the nodes in group $G_{j}$ if the Euclidian distance between $g_{i}$ and $g_{j}$ is smaller than $d_{\mathrm{th}}$. This means that if nodes from the group whose deployment point is far enough from the deployment point of *u’*s home group, the probability that they become *u*’s neighbors is small enough to be ignored. Thus, prior to deployment, for each group, the network deployer draws a circle centered at its deployment point with radius $d_{\mathrm{th}}$, records the deployment points that falls in such circle region on a *trust list*, and pre-loads the trust list into all the members of this group. Moreover, the network deployer uses a non-interactive public key establishment algorithm named SOK [26] to build pairwise keys shared between BS and sensor nodes. Let $k_{\mathrm{prv}}$ be the private key held by BS, and $k_{\mathrm{pub}}$ be the public key preloaded into sensor nodes. Using the private key $k_{\mathrm{prv}}$, the network deployer generates a certification $C_{k_{\mathrm{prv}}}\left(NID_{u}\|GID_{u}\right)$ on node *u*’s ID and group ID, and preloads the certification together with node *u*’s unique identity. In addition, BS shares a unique secret key with each node in the network, denoted by $k_{\langle i,\;\mathrm{BS}\rangle },\;i=1,\;2,\ldots ,M$. This shared secret key is also preloaded into the nodes before deployment.

Immediately after deployment, the beacon nodes located at the deployment points start to broadcast beaconing messages. The sensor nodes over the entire network keep on listening to the beaconing messages and recording the RSSI of the beaconing messages using the techniques from the Media Access Control layer protocols until the initiation phase ends. During the initiation phase, the nodes pick the largest three RSSI values and the corresponding deployment points. Meanwhile, the nodes start a neighbor discovery process and try to establish a unique pairwise key with each one of their immediate neighbors using some secret sharing techniques such as key pre-distribution [21,22]. Let $k_{\langle u,\;v\rangle }$ be the pairwise key shared between node *u* and *v*. During this process, the nodes also authenticate the integrity of their neighbors’ identities and belonging group by verifying the certification $C_{k_{\mathrm{prv}}}$ signed by BS. The nodes will rejects the forwarding requests by their neighbors which do not pass such authentication.

**Phase 2: Location Claim Generation and Probabilistic Forwarding.** Suppose that a node *u* in group $G_{i}$ receives a request from node *v* to forward a message. Node *u* first checks the group part of node *v*’s identity ($NID_{v}\|GID_{v}$) to make sure that node *v*’s identity is authenticated and its group ID is on the trust list. If so, node *u* accepts node *v* as a benign node and forwards the message as requested. Otherwise node *u* rejects to forward node *v*’s message and sends back a *Node Authentication Needed* (NAN) request to ask for authentication that proves node *v’*s integrity.

Upon receiving the NAN request, node *u* broadcasts a location authentication request (LAQ) around its neighborhood. The LAQ message consists of node *u*’s ID and corresponding timestamp, denoted by:

(13) $$LAQ=\langle {\mathrm{ID}}_{u}\|ts_{\mathrm{id}}\rangle .$$

After sending LAQ, node *u* start a timer $t_{\mathrm{wait}-LAR}$ to wait for its neighbors’ reply.

Once the neighbors $\left\{v_{1},\;v_{2},\;\ldots v_{r}\right\}$ of node *u* receive the LAQ request, they generate a message authentication code of their own IDs and corresponding timestamps using the key shared with BS. Then they encrypt all these data with the key shared with node *u* and send back the encrypted message to node *u.* We define this encrypted message as location authentication reply (LAR), represented as:

(14) $$\mathrm{LAR}=\langle enc_{k_{\langle v_{i},\;u\rangle }}\left({\mathrm{ID}}_{v_{i}}\|{\mathrm{MAC}}_{k_{{<}_{v_{i}},\;BS>}}\left({\mathrm{ID}}_{v_{i}}\right)\right)\rangle ,\;i=1,\;2,\;3,\;\ldots ,r,$$

where $ID_{v_{i}}$ represents the ID of the neighboring node $v_{i}$, $ts_{id_{v_{i}}}$ is a timestamp representing the time that LAR is sent back, $k_{\langle v_{i},BS\rangle }$ and $k_{\langle v_{i},\;u\rangle }$ represent the key shared between $v_{i}$ and BS, as well as between $v_{i}$ and *u*, respectively. The enc(⋅) and MAC(⋅) represent the encryption and MAC generation functions, respectively.

When the timer $t_{\mathrm{wait}-LAR}$ runs out, node *u* collects the received LAR messages, decrypts them, and extracts neighbors’ IDs and corresponding authentication tags from decrypted messages. Then node *u* obtains $\mathrm{IDs}=\left\{{\mathrm{ID}}_{v_{1}},\;{\mathrm{ID}}_{v_{2}},\;\ldots ,\;{\mathrm{ID}}_{v_{r}}\right\}$ and $\mathrm{Tags}=\left\{{\mathrm{Tag}}_{v_{1}},\;{\mathrm{Tag}}_{v_{21}},\;\ldots {\mathrm{Tag}}_{v_{r}}\right\}$, where ${\mathrm{Tag}}_{v_{i}}=\mathrm{MAC}\left({\mathrm{ID}}_{v_{i}}\right)$. Using the aggregated MAC approach, node *u* generates location claim request (LCQ), as $\mathrm{LCQ}=\langle L_{\mathrm{RSS}}\|IDs\|\mathrm{Tag}\rangle$. The $L_{\mathrm{RSS}}$ field contains the collected three largest RSSI and corresponding IDs of beacons. The IDs field contains the IDs of node *u*’s neighbors which reply *u*’s LAQ, as well as the ID of node *u* itself. The Tags field contains the authentication tags of IDs of node *u*’s neighbors, as well as the authentication tag of $L_{\mathrm{RSSI}}$ and node *u*’s ID. The detailed description of node *u*’s LCQ is as follows:

(15) $$\begin{array}{c} L_{\mathrm{RSS}}=\langle {\mathrm{RSSI}}_{x}\|{\mathrm{RSSI}}_{y}\|{\mathrm{RSSI}}_{z}\|g_{x}\|g_{y}\|g_{z}\rangle ,\\ \mathrm{IDs}=\langle ID_{u}\|ID_{v_{1}}\|{\mathrm{ID}}_{v_{2}}\|\ldots \|{\mathrm{ID}}_{v_{r}}\rangle ,\\ \mathrm{Tag}=\langle {\mathrm{MAC}}_{k_{\langle u,\;\mathrm{BS}\rangle }}\left(L_{RSSI}\|{\mathrm{ID}}_{u}\right)⊕\left(\overset{r}{\underset{i=1}{⊕}}{\mathrm{MAC}}_{k_{\langle v_{i},\mathrm{BS}\rangle }}\left({\mathrm{ID}}_{v_{i}}\right)\right)\rangle ,\\ \mathrm{LCQ}=\langle L_{\mathrm{RSS}}\|IDs\|\mathrm{Tags}\rangle . \end{array}$$

Note that the reason we add node *u*’s ID and corresponding authentication tag is to prevent the replay and forgery attacks.

After the task of LCQ generation, node *u* selects several nodes from its neighbors in IDs fields to forward the LCQ message. When the selected neighbors receive the LCQ from node *u*, they check whether its ID is on the IDs filed. If so, they forward the LCQ message to BS with the probability $P_{f}$, otherwise directly discard such message.

**Phase 3: Replica Detection and Revocation.** Upon receiving LCQ message from node *u*, BS computes $\mathrm{Ta}g^{\prime }={\mathrm{MAC}}_{k_{\langle u,\;\mathrm{BS}\rangle }}\left(L_{RSSI}\|{\mathrm{ID}}_{u}\right)⊕\left(\overset{r}{\underset{i=1}{⊕}}MAC_{k_{\langle v_{i},\;\mathrm{BS}\rangle }}\left({\mathrm{ID}}_{v_{i}}\right)\right)$ using the key shared with node *u* and its neighbors $v_{1},\;v_{2},\;\ldots ,$ $v_{r}$ to find out whether $Tag^{\prime }$ equals to Tag, so as to verify the integrity of $L_{RSS}\|IDs$. If LCQ does not pass integrity verification, BS directly discards the LCQ message. Otherwise, BS parses the data payload $L_{RSS}\|\mathrm{IDs}$ and accordingly decides whether node *u* is replica in the following steps.

**Step 1: DNV extraction.** Using the RSSI distance measurement model proposed in [27], BS can derive node *u*’s distance to the corresponding beacon nodes. Let constant *A* be the signal strength (in dBm) received at the point that 1 meter away from the signal source, and *r* be multipath fading factor. Both *A* and *r* are regarded as the prior network environment knowledge. Given the RSSI $P_{R}$ (in dBm) about the signal source, the distance *d* between receiver and source is obtained by:

(16) $$\mathrm{lg}d\;=\;-\frac{P_{R}-A}{10r}.$$

**Figure 4:**
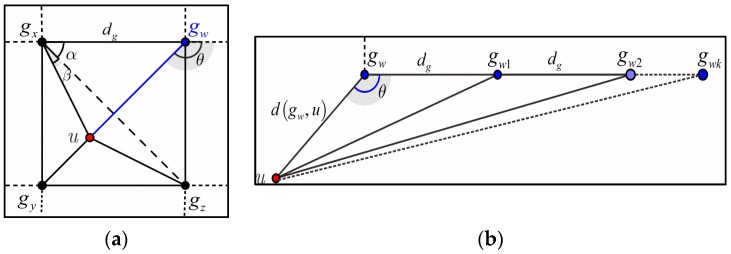
Derivation of DNV by the RSSI data from the $L_{\mathrm{RSS}}$ field of LCQ. (**a**) Deciding node *u*’s position in a single grid using RSSI of the nearest three deployment points; and (**b**) derivation of the distances between node *u* and the deployment points using the group deployment knowledge.

According to Equation (16), we can derive the distance from node *u* to deployment point $g_{x}$ (respectively, $g_{y},\;g_{z}$), denoted by $d\left(g_{x},\;u\right)$ (respectively, $d\left(g_{y},\;u\right)$, $d\left(g_{z},\;u\right)$). The deployment points $g_{x},\;g_{y},\;g_{z}$ are the nearest to node *u*. Based on the triangle axiom, it is easy to infer that node *u* is located in a triangular region of which the vertexes are $g_{x},\;g_{y},\;g_{z}$, as illustrated in Figure 4a.

Given the distances $d\left(g_{x},\;u\right)$, $d\left(g_{y},\;u\right)$, $d\left(g_{z},\;u\right)$, and angle α=π/4, the angle β can be obtained by:

(17) $$∡\beta =\arccos\left(\frac{2d_{g}^{2}+d^{2}\left(g_{x},u\right)-d^{2}\left(g_{z},\;u\right)}{2d\left(g_{x},\;u\right)d\left(g_{z},\;u\right)}\right).$$

Based on the primary geometric theory, we can derive the distance between node *u* and deployment point $g_{w}$:

(18) $$d\left(g_{w},\;u\right)=\sqrt{d^{2}\left(g_{x},u\right)+d_{g}^{2}-2d_{g}d\left(g_{x},u\right)\cos\left(\beta +\frac{\pi }{4}\right)},$$

and the radian value of angle θ:

(19) $$∡\theta =\pi -\arccos\left(\frac{d_{g}^{2}-d\left(g_{x},u\right)d_{g}\cos\left(\beta +\frac{\pi }{4}\right)}{d\left(g_{w},u\right)d_{g}}\right).$$

Consider that the deployment point $g_{w1},\;g_{w2},\;\ldots ,\;g_{wk}$ that in the three o’clock direction of $g_{w}$, as shown in Figure 4b, the distance between node $g_{wi}$ and $g_{w}$ is $i\cdot d_{g},\;i=1,\;2,\;\ldots ,k$. Given the distance $d\left(g_{w},\;u\right)$, $d\left(g_{wi},\;g_{w}\right)$, and the angle θ, we can obtain the distance from node *u* to the nodes $\left\{g_{i}|i=1,\;2,\;\ldots ,\;k\right\}$ as:

(20) $$d\left(g_{wi},\;u\right)=i^{2}d_{g}^{2}+d^{2}\left(g_{w},u\right)-2i\cdot d_{g}\cdot d\left(g_{w},u\right)\cos\theta ,\;i=1,\;2,\ldots ,k$$

By using similar methodology, BS can obtain the distance between node *u* and other deployment points in the target region. Let $Z=\left\{z_{g_{i}}=d\left(g_{i},\;u\right)|i=1,\;2,\;\ldots m\right\}$ denote such distances to the deployment points over the entire network. According to Inference 1, BS can derive the DNV with respect to location information $L_{RSS}$ based on set *Z*, denoted by:

(21) $$S_{nei-D}\left(u\right)=\frac{\left(\mu_{L_{u}}^{\left(G_{1}\right)},\;\mu_{L_{u}}^{\left(G_{2}\right)},\ldots ,\mu_{L_{u}}^{\left(G_{m}\right)}\right)}{\sum_{i=1}^{m}\mu_{L_{u}}^{\left(G_{i}\right)}},\;\mu_{L_{u}}^{\left(G_{i}\right)}=\frac{M}{m}g\left(z_{g_{i}}|k\in G_{i}\right),\;i=1,\;2,\;\ldots ,\;m.$$

**Step 2: Conflict detection.** Using the method in Step 1, BS derives the distance between node *u* and deployment point $g_{u}$. If the value of distance $d\left(u,\;g_{u}\right)$ is larger than a threshold $\tau_{\mathrm{CD}}$, BS will determine node *u* as replica. Furthermore, BS searches the cached location claims that pass the detection to find out whether it has received a former version of node *u’*s location claim. If so, BS compares the $L_{\mathrm{RSS}}$ fields of the two versions to check whether a conflict exists. In the case that there exists a conflict, BS removes the conflicted location claim from cache and start the replica revocation operations in Step 3.

**Step 3: Decision and revocation.** BS parses the IDs field, and counts the IDs of node *u*’s neighbors by groups to which they belong. Then BS obtain a observed neighboring vector (ONV), which indicates the *u*’s neighboring distribution observed by BS. Note that the ONV is generated by node *u*’s neighbors and its integrity is verified by BS, which means that it has high reliability. Let $S_{ob}\left(u\right)=\left\{S_{ob}^{\left(G_{1}\right)}\left(u\right),\;S_{ob}^{\left(G_{2}\right)}\left(u\right),\;\ldots ,\;S_{ob}^{\left(G_{m}\right)}\left(u\right)\right\}/\sum_{i=1}^{m}S_{ob}^{\left(G_{i}\right)}\left(u\right)$ denote the node *u*’s ONV. BS computes the NV-LS between the $S_{ob}\left(u\right)$ and $S_{nei-D}\left(u\right)$. BS firstly encodes $S_{ob}\left(u\right)$ and $S_{nei-D}\left(u\right)$ using LSH coding algorithms like MinHash [12], of which the output are *b-*bit binary sequence. BS then computes the Hamming distance $D_{h}$ of the two output sequences, and finally obtain the NV-LS as:

(22) $$\mathrm{sim}\left(S_{nei-D}\left(u\right),\;S_{ob}\left(u\right)\right)=1-\frac{D_{h}\left(LSH\left(S_{nei-D}\left(u\right)\right),\;LSH\left(S_{ob}\left(u\right)\right)\right)}{b}.$$

If the NV-LS is larger than a threshold $\tau_{\mathrm{RD}}$, BS determines node *u* as benign and add the $L_{\mathrm{RSS}}$ field of node *u*’s location claim to cache of BS. Otherwise, it determines node *u* as replica and raises an alarm. After the decision is reached, BS scans $S_{ob}\left(u\right)$ again to find the non-zero elements which indicates the groups whose members reside in node *u*’s neighborhood, and directed broadcasts the location claim decision (LCD) messages to such groups. The LCD can be represented as:

(23) $$\mathrm{LCD}=\langle ID_{u}\|\mathrm{RES}\|{\mathrm{Sig}}_{k_{\mathrm{priv}}}\rangle ,$$

where the field RES is the detection result about node *u*, and ${\mathrm{Sig}}_{k_{priv}}$ is the digital signature generated by BS with its private key. BS send several copies of LCD message to ensure that the LCD message reaches the node *u*’s neighboring groups. Once such message reaches one member of the target group, it will be flooded throughout the entire group. The cost of such local flooding is limited because it happens in a very small region. This ensures that every member in the target groups receives the LCD message. The nodes in the target groups add node *u* to their conditional trust lists and start to forward the messages from node *u* when the LCD message indicates that node *u* is benign.

### 4.4. Obtaining the Replica Detection Threshold

In this work, we adopt a training approach to estimate the replica detection threshold $\tau_{\mathrm{RD}}$, and obtain the training data from network simulations. According to the deployment strategy presented in Section 3.2, we generate a network simulation scenario, deploy our proposed protocol and repeat the simulation for *k* rounds. In each round of simulation, we obtain the training data as follows:

**Step** **1.** We obtain the actual physical position $L_{a}\left(i\right)=\left(x_{ai},\;y_{ai}\right)$, where i=1, 2, …, N, and derive the actual DNV for the selected nodes, represented as:
  (24) $$S_{nei-D}^{a}=\left\{S_{nei-D}^{a}\left(i\right)|\frac{\left(\mu_{L_{a}\left(i\right)}^{\left(G_{1}\right)},\;\mu_{L_{a}\left(i\right)}^{\left(G_{2}\right)},\ldots ,\mu_{L_{a}\left(i\right)}^{\left(G_{m}\right)}\right)}{\sum_{i=1}^{m}\mu_{L_{a}\left(i\right)}^{\left(G_{i}\right)}},\;i=1,\;2,\;\ldots ,\;N\right\}$$
**Step** **2.** We collect the $L_{\mathrm{RSS}}$ field of LCQ message from the *N* selected nodes, and compute the corresponding estimated position $L_{e}\left(i\right)=\left(x_{ei},\;y_{ei}\right)$ where i=1, 2, …, N. Then we derive the estimated DNV for the selected nodes, represented as:
  (25) $$S_{nei-D}^{e}=\left\{S_{nei-D}^{e}\left(i\right)|\frac{\left(\mu_{L_{ei}}^{\left(G_{1}\right)},\;\mu_{L_{ei}}^{\left(G_{2}\right)},\ldots ,\mu_{L_{ei}}^{\left(G_{m}\right)}\right)}{\sum_{i=1}^{m}\mu_{L_{ai}}^{\left(G_{i}\right)}},\;i=1,\;2,\;\ldots ,\;N\right\}$$
**Step** **3.** We collect the IDs field of the LCQ message from the selected nodes, and derive the ONV for them: $S_{ob}=\left\{S_{ob}\left(i\right)|\left(S_{ob}^{\left(G_{1}\right)}\left(i\right),\;\ldots ,\;S_{ob}^{\left(G_{m}\right)}\left(i\right)\right),\;i=1,\;2,\;\ldots ,\;N\right\}$.
**Step** **4.** We derive the NV-LS of $L_{a}$ and $L_{e}$, denoted by $sim_{a}$ and ${\mathrm{sim}}_{e}$, respectively, where ${\mathrm{sim}}_{a}=\left\{sim_{a}\left(i\right)|sim\left(S_{nei-D}^{a}\left(i\right),\;S_{ob}\left(i\right)\right),\;i=1\;\ldots N\right\}$, and ${\mathrm{sim}}_{e}=\left\{sim_{e}\left(i\right)|sim\left(S_{nei-D}^{e}\left(i\right),\;S_{ob}\left(i\right)\right),\;i=1\ldots N\right\}$. Then we can compute the deviation $\chi_{sim}$ between $sim_{a}$ and ${\mathrm{sim}}_{e}$ caused by the network uncertainties and measurement errors, represented as:
  (26) $$\chi_{\mathrm{sim}}=\left\{\chi_{sim}\left(i\right)|\left|sim_{a}\left(i\right)-{\mathrm{sim}}_{e}\left(i\right)\right|,\;i=1,\;2,\;\ldots ,\;N\right\}$$

After *k* round repeated simulations, we obtain *k* sample sequence $\chi_{\mathrm{sim}}$, i.e., k×N samples of the NV-LS deviation as the training data, which form a sample distribution. Then we use the ξ-percentile to decide the threshold from these training data, which means that 1−ξ percent of the samples falls within the range $\left(\tau_{RD},\;1\right)$ if $\tau_{\mathrm{RD}}$ equal to the value of the ξ-percentile.

## 5. Security Analysis

Unlike other security threats against WSNs, such as node compromise and forgery, thorough elimination of replica nodes in a sensor network is infeasible and uneconomic since there are several choices for the adversary to hide the existence of replicas from the detection schemes at the cost of minimizing the functionality of replicas. For instance, the adversary can place the replica nodes very close to its original compromised ones, or strengthen the replicas’ transmission power towards the original ones’ neighbors, to make the replicas act exactly the same as its original compromised nodes. In this way, it is extremely difficult to find out which node is a replica, although the adversary benefits very little from such a kind of node replica attack. Consequently, in the security analysis of this section, we concentrate on the investigation of the effectiveness of our scheme that suppresses the damage caused by the replicas of a given compromised node.

To evaluate the security performance of our proposal, we adopt an 80-bit security level (RSA-1024 equivalent) elliptic curve Diffie-Hellman (ECDH) scheme [28] to provide a security guarantee for the key establishment during the process of neighbor discovery. We also adopt an 80-bit security level Data Encryption Standard (DES) algorithm [29] for the LAR message transmission.

### 5.1. Limitation of the Impact Range of Node Replica Attack

We first define the number of ordinary nodes that accept the replicas and forward their messages as the metric to quantify the damage of the node replica attack. Suppose the adversary compromises a node *v* and scatters several replicas of node *v* in the target deployment region. Under the protection of our scheme, we can observe that only the nodes which have node *v*’s group ID in their trust lists will accept the replicas as trusted neighbors. Recall that the length of the trust list depends on the value of $d_{th}$. We can accordingly derive the upper bound of the impact of node replica attack with respect to $d_{\mathrm{th}}$. We can infer that the nodes are impacted by node *v*’s replicas if and only if the deployment points of their home groups falls in the circle region centered at node *v* with the radius $d_{th}$. Then the upper bound $\varsigma_{imp}^{\left(\mathrm{upper}\right)}$ of the number of nodes impacted by node *v*’s replicas can be obtained by:

(27) $$\varsigma_{imp}^{\left(\mathrm{upper}\right)}=\frac{M}{m}{\left\|\left\{g_{i}|dist\left(g_{u},\;g_{i}\right)=d_{g}\sqrt{k^{2}+r^{2}}\leq d_{\mathrm{th}},\;0<k,\;r\leq \left\lfloor \frac{\sqrt{m}}{2}\right\rfloor \right\}\right\|}_{0}.$$

**Figure 5:**
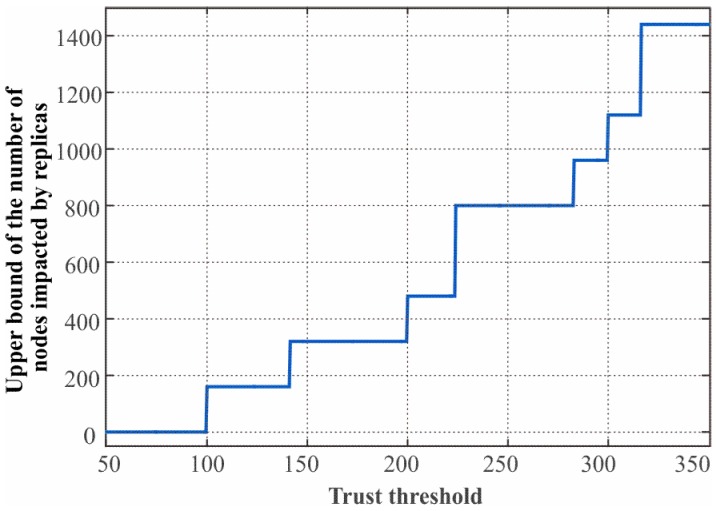
The trust threshold $d_{th}$ vs. the upper bound of replica impact $\varsigma_{\mathrm{upper}}$ given $M=2560,\;m=64,\;g_{d}=100$.

To further investigate the impact of node replica attack under the limitation $d_{\mathrm{th}}$ of our scheme, we set an ideal scenario in which M=2560 nodes are deployed in $700\times 700\;m^{2}$ area with *m* = 64 groups, the distance between deployment points $d_{g}=100\;m$, and the communication radius of sensor nodes and beacon nodes is 150 m. The numeric result about Equation (27) are shown in Figure 5. We observe that the upper bound of replica impact step up with the increase of $d_{\mathrm{th}}$. That’s what we expect since more groups are involved in the replicas’ range when the trust threshold rises. When $d_{th}$ falls in the range (100, 150), about 6.25% of nodes are affected by the replicas. When $d_{th}$ falls in the range (150, 200), the fraction of the affected nodes rise to 12.5%. This is still a small fraction considering the very large impact range of replica attack. Thus, we should set $d_{\mathrm{th}}$ to a small value so as to suppress the impact of such a replication attack. However we would like to point out that the network connectivity and communication efficiency will be severely degraded when the value of $d_{th}$ is very small since the node will keep discarding the messages from the nodes whose group IDs are not on the trust list, as shown in the analysis of next section.

### 5.2. Defense Capacity Analysis on Location Claim-Based Detection

To compensate for the degradation of the network connection caused by the trust-based selective forward, our scheme gives the nodes whose messages are rejected by their neighbors a second chance to prove their integrity by a neighboring vector-based location claim. However, we infer that the adversary can still take at least three potentially effective attack strategies against our scheme. We analyze the defense capacity under the three attack strategies respectively in this subsection.

**Strategy I: GID forgery.** In this strategy, the adversary modifies the GID of the replicas so that the replicas’ neighbors will accept them as nodes from trusted groups, and forward their messages. However recall that the integrity of GID is protected by the certification $C_{k_{prv}}\left(NID\|GID\right)$ signed by BS, as well as the BS being assumed to be a trusted entity, which means that its private key cannot be compromised. Thus, to achieve the modification of GID, the adversary has to compromise the public key certification algorithm. In this case, the defense capacity of this work depends on the security strength of the adopted certification algorithm itself, which is beyond our discussion. In other words, if the adopted certification algorithm provides enough security strength, our scheme can defeat such an attack strategy.

**Strategy II: *L*_RSS_ forgery.** In this strategy, the adversary generates falsified $L_{\mathrm{RSS}}$ field of LCQ according to the neighboring distribution derived from the IDs field so as to bypass the neighboring vector-based replica detection at BS. This means that the value of $L_{RSS}$ should be kept consistent with the true position of replicas. The replicas, thereby, should be placed at the position less than $\tau_{CD}$ away from the deployment point of their home group, otherwise they will be caught by the conflict detection of BS. This constrains the adversary’s benefits when the threshold $\tau_{CD}$ is small. For instance, when $\tau_{\mathrm{CD}}=R_{z}$, the replicas and their origin compromised node should be deployed in the same group under the limitation of our scheme, which gains little benefits than that of simply performing node compromise attack. However according to the deployment model used in this work, the node deployment is not accurate but follows two-dimension Gaussian distribution. Several nodes locate at the positions outside the communication range of deployment points with probability, resulting in the false positives in our work. Let $P_{a}$ be the false positive rate, i.e., the probability that the benign nodes are determined as replicas given they are located outside their home group. Then we have:

(28) $$\begin{array}{cc} P_{a} & =1-\int_{0}^{2\pi }\int_{0}^{\tau_{\mathrm{CD}}}f\left(\rho \cos\theta ,\;\rho \sin\theta \right)\cdot d\rho d\theta \\ & =1-\int_{0}^{2\pi }\int_{0}^{\tau_{\mathrm{CD}}}\frac{1}{2\pi \sigma^{2}}\exp\left(-\frac{\rho^{2}}{2\sigma^{2}}\right)\cdot d\rho d\theta \\ & =\exp\left(-\frac{\tau_{\mathrm{CD}}^{2}}{2\sigma^{2}}\right), \end{array}$$

where σ is the standard deviation of the two-dimension Gaussian distribution, and the indicator about deployment accuracy in our context.

**Figure 6:**
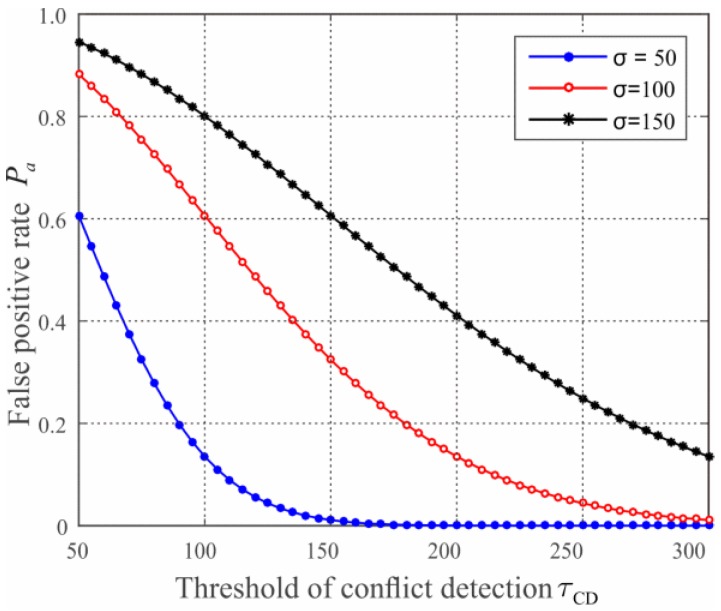
The conflict detection $\tau_{\mathrm{CD}}$ vs. false positive rate $P_{a}$ given σ=50, 100, 150, respectively.

Using the same simulation scenario in Section 5.1, we consider three different cases in which σ=50, 100, 150, respectively. In each case, we vary $\tau_{\mathrm{CD}}$ from 50 to 300, the numeric results about the false positive rate of our scheme are shown in Figure 6. We observe that the false positive rate $P_{a}$ decreases significantly with the increase of the conflict detection threshold $\tau_{\mathrm{CD}}$. This is reasonable since more suspected nodes pass the conflict detection when the value of conflict detection threshold is larger. We also observe that $P_{a}$ increases with the rise of σ when $\tau_{CD}$ is fixed, which indicates that the security performance of our scheme enhances when the deployment accuracy improves. When σ=50, $\tau_{\mathrm{CD}}=R_{z}=150$, the value of false positive rate is less than 1%, which means that the proposed scheme achieves a promising performance when the parameter are properly configured.

**Strategy III: IDs forgery.** We assume that the adopted aggregated MAC and encryption algorithms provide enough security strength for our scheme to protect the integrity and non-repudiation of LAR message. This prevents the adversary from misleading the replica decision of BS by generating falsified LAR messages. However the adversary still has a chance to drive the compromised nodes to modify the IDs field. The replica nodes can deliberately remove several neighbors’ identities in the IDs field of LCQ message. For a particular replica node *v*, it can remove the identities of neighbors belonging to node *v*’s nearest groups, denoted by $G_{v1},\;\ldots G_{vk}$, so as to reduce the corresponding elements $S_{\mathrm{ob}}^{\left(G_{v1}\right)},\;\ldots ,\;S_{\mathrm{ob}}^{\left(G_{vk}\right)}$ of node *v*’s ONV $S_{ob}\left(v\right)$. Note that since node *v* cannot add falsified IDs into the IDs field, the value of $S_{\mathrm{ob}}^{\left(G_{v1}\right)},\;\ldots ,\;S_{\mathrm{ob}}^{\left(G_{vk}\right)}$ should be reduced to a very low level if node *v* is far away from its origin. Recall that because node *v* has to select neighbors in the IDs field for message forwarding, the behavior of removing a neighbors’ identity from the IDs field results in a significant decrease in the success probability that node *v*’s LCQ message arrives at BS. Hence, the negative ID forgery is limited by the probabilistic LCQ forwarding mechanism.

## 6. Performance Evaluation

In this section, we evaluate the performance of our proposed scheme from both an effectiveness and efficiency perspective. Firstly, we theoretically analyze the efficiency of our proposed scheme in terms of the communication, computation, and storage overheads, and then provide further quantitative evaluation on the effectiveness of our scheme through simulation experiments. Furthermore, we compare the performance of our scheme with the previous replica detection approaches [6,11].

### 6.1. Communication, Computation, and Storage Overhead

**Communication overhead.** In our context, the communication overhead is defined as the extra traffic brought by our scheme. We consider a worst case in which the trust threshold $d_{th}$ is set to a small value so that the nodes will be asked for location claims if they are placed outside the range of its home group. According to Equation (28), the number to nodes that are required to forward location claims is $M\cdot P_{a}=M\cdot \exp\left(-\frac{\tau_{\mathrm{CD}}}{\sigma^{2}}\right)$. For a particular node *v*, the average number of its neighbors is $N_{v}=\frac{M}{m}\sum_{i=1}^{m}g\left(dist\left(L_{v},\;L_{g_{i}}\right)|k\in G_{i}\right)$. During the process of node *v*’s location claim, *r* out of node *v*’s neighbors ask node *v* for node authentication, node *v* sends LAQ to all its $N_{v}$ neighbors, receives, at most, the same amount of LAR from those neighbors, and then forwards the LCQ to BS. Finally BS forwards LCD to the target groups. According to [6], the average hops between two randomly-selected nodes is approximately $O\left(\sqrt{N}\right)$. Thus, we can derive the communication overhead for a particular node as:

(29) $$c_{comm}=r\cdot l_{\mathrm{NAN}}+N_{v}\cdot \left(l_{LAQ}+L_{LAR}\right)+O\left(\sqrt{M}\right)\cdot l_{\mathrm{LCQ}}+k\cdot O\left(\sqrt{M}\right)\cdot l_{\mathrm{LCD}},$$

where $l_{NAN},\;l_{\mathrm{LAQ}},\;l_{\mathrm{LAR}},\;l_{LCQ},\;l_{\mathrm{LCD}}$ are the message length of NAN, LAQ, LAR, LCQ, and LCD, respectively, whereas *k* is the number of nonzero elements of $S_{\mathrm{ob}}$.

Then we have the upper bound of total communication overhead, as follows:

(30) $$\begin{array}{cc} C_{comm} & =M\cdot P_{a}\cdot \left(r\cdot l_{\mathrm{NAN}}+{\bar{N}}_{\mathrm{nei}}\cdot \left(l_{LAQ}+L_{LAR}\right)+O\left(\sqrt{M}\right)\cdot l_{\mathrm{LCQ}}+k\cdot O\left(\sqrt{M}\right)\cdot l_{\mathrm{LCD}}\right)\\ & =\;O\left(P_{a}\cdot \left(M{\bar{N}}_{\mathrm{nei}}+M\sqrt{M}\right)\right), \end{array}$$

where ${\bar{N}}_{\mathrm{nei}}$ is the average number of neighbors for a node. From Equation (30), we can see that the upper bound of communication overhead in the worst case depends on the total number of nodes in the entire network and average number of neighbors.

**Computation overhead.** Since, in our scheme, the cryptographic operations consume the overwhelming majority of the computation resources, we use the average number of cryptographic operations as a metric to measure the computation overhead for our scheme. We assume that BS is a trusted entity with strong computation and storage capacity, so we only focus on the computation overhead that incurs at the ordinary sensor nodes. Let $Q_{cert}$ denote the computation overhead for the single-time operation of certification verification, let $Q_{\mathrm{MAC}}$ denote the overhead for the single-time operation of MAC generation, and let $Q_{\mathrm{ENC}}$ denote the overhead for the single-time operation of message encryption. Besides, we assume that $f_{c}$ is the fraction of the nodes are replicas, and they are placed randomly following a two-dimensional uniformly distribution. For a particular node *u*, it has to verify the certifications of their neighbors’ IDs $N_{u}$ times during neighbor discovery, where $N_{u}$ is the number of node *u*’s neighbors. Node *u* also has to generate the MAC and encrypt the LAR message for the nodes needed to be location authenticated. Note that. in the worst case, there are $N_{u}\cdot f_{c}$ replicas and $P_{a}\left(1-f_{c}\right)N_{u}$ benign nodes are asked for location claims. Finally, in the case that node *u* is asked for location claims (the case incurs with the probability $f_{c}+\left(1-f_{c}\right)P_{a}$), node *u* generates an extra MAC of its own ID for LCQ message. Then we obtain the computation for a particular node by:

(31) $$c_{\mathrm{cmp}}={\bar{N}}_{\mathrm{nei}}Q_{cert}+{\bar{N}}_{\mathrm{nei}}\left(f_{c}+\left(1-f_{c}\right)P_{a}\right)\left(Q_{MAC}+Q_{\mathrm{enc}}\right)+\left(f_{c}+\left(1-f_{c}\right)P_{a}\right)Q_{MAC}.$$

The total computation overhead for our scheme is accordingly:

(32) $$\begin{array}{cc} C_{\mathrm{cmp}} & =M{\bar{N}}_{nei}Q_{\mathrm{cert}}+M\left({\bar{N}}_{\mathrm{nei}}+1\right)\left(f_{c}+\left(1-f_{c}\right)P_{a}\right)Q_{MAC}+M\left(f_{c}+\left(1-f_{c}\right)P_{a}\right)Q_{\mathrm{enc}}\\ & =O\left({\bar{N}}_{\mathrm{nei}}\left(f_{c}+\left(1-f_{c}\right)P_{a}\right)+\left(f_{c}+\left(1-f_{c}\right)P_{a}\right)\right). \end{array}$$

**Storage overhead.** As aforementioned, we focus on the storage occupation of our scheme on the resource-constrained sensor nodes. Recall that the conflict detection and replica detection are performed at BS, thus, the sensor nodes in the network do not need to cache other nodes’ location claims. In this work, the nodes only keep the secret key and certification signed by BS in memory, whose size is negligible compared to that of location claims.

**Comparison of the resource efficiency**. We compare the resource efficiency with the prior works [6,14] in terms of communication, computation, and storage overhead. The corresponding comparison results are shown in Table 2, Table 3 and Table 4. We present the results on the additional traffic incurred by the schemes all over the network in Table 2, the results on the average computation overhead on each node in the network in Table 3, and the results on the storage occupation for the location claims in Table 4.

As illustrated in Table 2, Table 3 and Table 4, the overhead of the line-selected and randomized multicast scheme in [6] linearly increases with the rounds of location claim over the network lifetime, which means that the overhead of these schemes will exceed the other reference schemes as time increases. The scheme I in [11] is clearly the most resource efficient since it hardly brings any additional tasks to network routines for replica detection. However, this scheme only provides primary security services and will be defeated if the replica nodes modify their group ID to bypass the trust threshold-based detection of scheme I. Compared with scheme I, the scheme II and III in the same paper provide much better security performance at the cost of extra overhead incurred by location claims. However those two schemes still cannot resist the attack strategy in which the replicas provide the falsified location claims that exactly the same as their original compromised nodes. In contrast, our scheme can effectively resist the falsified group ID and falsified location claim attack strategies. The communication overhead of our scheme is slightly higher than that of Scheme II, and much lower than that of Scheme III, given that the average number of neighbors ${\bar{N}}_{nei}$ is much less than the total number of nodes *M* in most cases, whereas the computation and storage overhead of our scheme is much lower than similar schemes including Scheme II and III. This is because, unlike Scheme II and III, our scheme is able to protect integrity of the location claims without the en-route digital signature used in the two schemes. Additionally, the conflict check and replica detection in our scheme is performed at BS, which significantly reduces the computation overhead and storage occupation on the resource-constraint sensor nodes.

From the analysis above, we believe that compared with the prior works [6,11], our proposed scheme enhances the security resilience to the various attack strategies at the cost of a reasonable increase of communication overhead. Additionally, our work achieves promising performance in terms of computation and storage overhead. This is because the major computation tasks are deployed on BS.

### 6.2. Experimental Setup and Methodology

To validate the previous theoretical analysis results, we conduct experiments on the TOSSIM [30] platform to evaluate effectiveness and efficiency in terms of the detection rate and communication overhead under various network configuration. Based on the experiment results, we compare the performance of the proposed scheme with the previous discussed works, namely the randomized multicast and line-selected multicast schemes in [6], as well as Schemes I–III in [11].

In this simulation, following the network deployment strategy in Section 3.2, we establish a simulation scenario by placing *M* sensor nodes, which is averaged to *m* = 64 groups, in a 700 × 700 m^2^. The target area are divided into a 8 × 8 mesh grid, and the deployment points of each group are placed at the cross points of such grid. The group members are deployed following the two-dimension joint Gaussian distribution, where the mean is the coordination of corresponding deployment point while the standard deviation is σ. We set the maximum communication radius $R_{z}=150$ m, and the distance between two neighboring deployment points $d_{g}=100$ m. We assume that the target area is located in an open space with a low-level asymmetric radio channel, and choose the corresponding simulation parameters as shown in Table 5.

To emulate the three aforementioned attack strategies, we take the following procedure:

**Step** **1.** After deployment, we randomly pick *k* (k=⌊0.005×M⌋) nodes from the network topology, and mark them as compromised nodes. Let $L_{c1},\;L_{c2},\;\ldots ,\;L_{ck}$ denote the locations of these *k* compromised nodes.
**Step** **2.** For each compromised node, we generate *r* replica nodes and place them *D* meters away from their original compromised nodes. $L_{p1}^{ci},\;L_{p2}^{ci},\ldots L_{pr}^{ci}\;$ denote the locations of compromised node *i*’s (*i* = 1, 2, …, *k*) replicas, where $\left|L_{pj}^{ci}-L_{ci}\right|=D$.
**Step** **3.** In attack strategy I, the replica node modifies its group identity GID to the nearest group while keeping its NID the same with its origins. In attack strategy II, the replica nodes keep their $L_{\mathrm{RSS}}$ field consistent with the IDs field in their LCQ message. In attack strategy III, the replica nodes make their $L_{\mathrm{RSS}}$ field the same with their original compromised nodes, and keep their IDs field consistent with the $L_{\mathrm{RSS}}$ field in the LCQ message.

Moreover, in order to investigate the performance of the proposed scheme under different environments, we adopt a variety of network configurations by varying the deployment standard deviation σ from 50 to 150, the number of replicas for each original compromised node *r* from one to five, as well as the distance between replicas and origins D from 150 to 300. We use two metrics to evaluate the proposed scheme: (1) detection rate: suppose there are *m* replica nodes in the network, and *n* of them are detected, the detection rate is measured as n/m; and (2) average communication overhead: during the entire lifetime of the simulation, the average packets of the proposed scheme sent per node. For each network configuration, we conduct our simulation for 6000 s, and repeat the simulation 100 times, using the average value of the above metrics as the experiment results.

### 6.3. Results and Discussion

**Figure 7:**
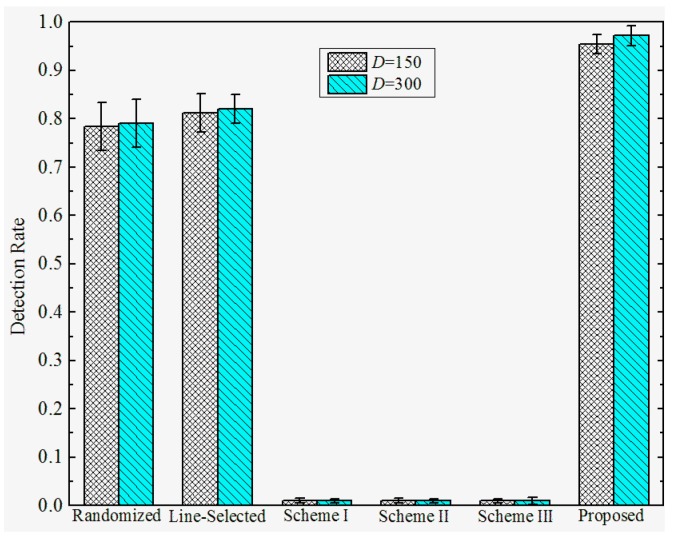
Detection rate of the proposed versus prior works under attack strategy I when σ=100.

**Figure 8:**
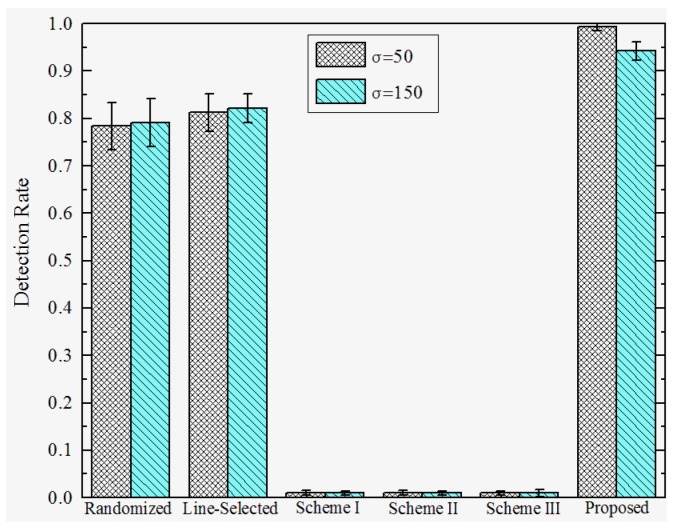
Detection rate of proposed versus prior works under attack strategy I when *D* = 200.

**Detection Rates under Attack Strategy I.** To investigate the effectiveness under various attack strategies, we present the detection rate of the proposed and prior works under different *D*, and σ. We set the total number of sensor nodes M=2560, the number of compromised nodes k=12, and the number of replicas for each compromised node r=3. The detection rates of the proposed and previous works under attack strategy I are shown in Figure 7 and Figure 8. We observe that the Scheme I–III have almost no resilience to attack strategy I, since the adversary can make sensor nodes accept the replicas as trusted neighbors by falsifying the GID field of the replicas’ message. In this case, Schemes I–III will not trigger the location claim detection mechanism. On the other hand, our proposed scheme and the randomized and line-selected multicast can effectively resist such attack strategies since all of these schemes do not rely on the trusted neighbor detection mechanism. Furthermore, our proposed scheme achieves a promising effectiveness and robustness under different *D* and σ, the detection rate comes to around 97% in all of the network configurations. This is because, in our scheme, the integrity of GID is guaranteed by the certification signed by BS, which can hardly be compromised by the adversary.

**Figure 9:**
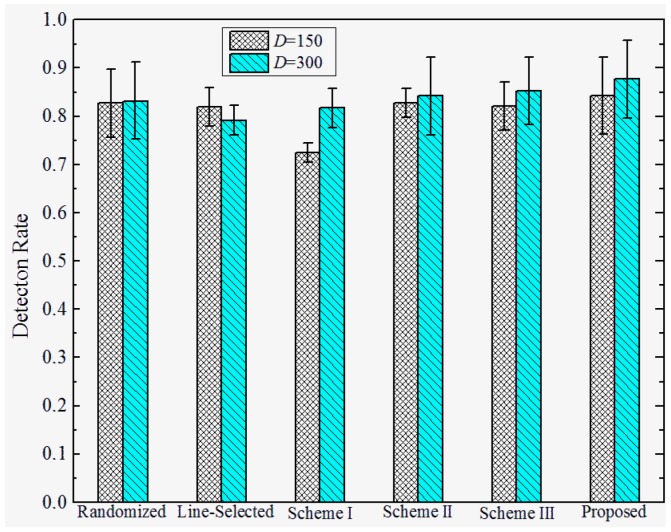
Detection rate of the proposed versus prior works under attack strategy II when σ=100.

**Figure 10:**
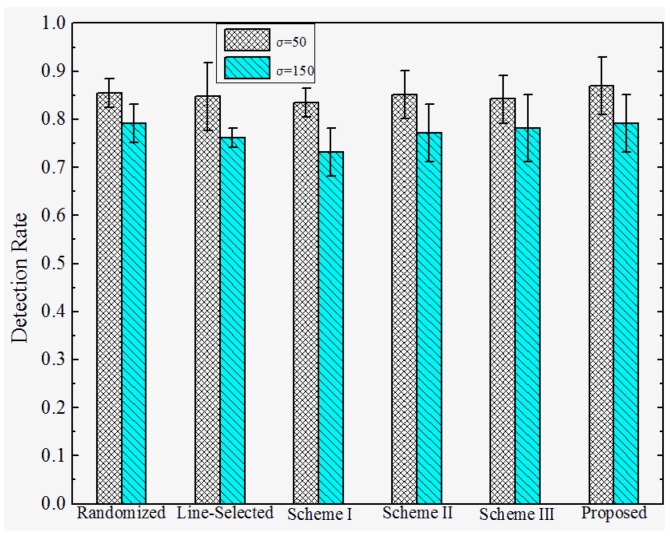
Detection rate of the proposed versus prior works under attack strategy II when *D* = 200.

**Detection Rate under Attack Strategy II**. The detection rates under attack strategy II are shown in Figure 9 and Figure 10. We observe that both the proposed and previous works can achieve relatively high detection rates against attack strategy II. This is because they adopt similar location claim confliction detection mechanisms. Furthermore, the detection rates of all the schemes rise when the distance *D* between replicas and their origins becomes higher. We infer that is because the deviation between the replicas and their origins becomes larger, resulting in higher detection rates, whereas the detection rates of all of the schemes decrease as the standard deviation σ increases. This means that the detection rates are hindered by the deployed accuracy.

**Figure 11:**
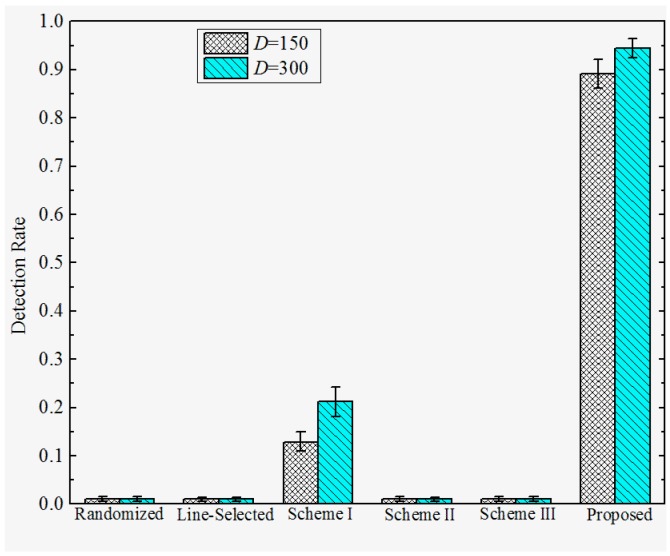
Detection rate of the proposed versus prior works under attack strategy III when σ=100.

**Figure 12:**
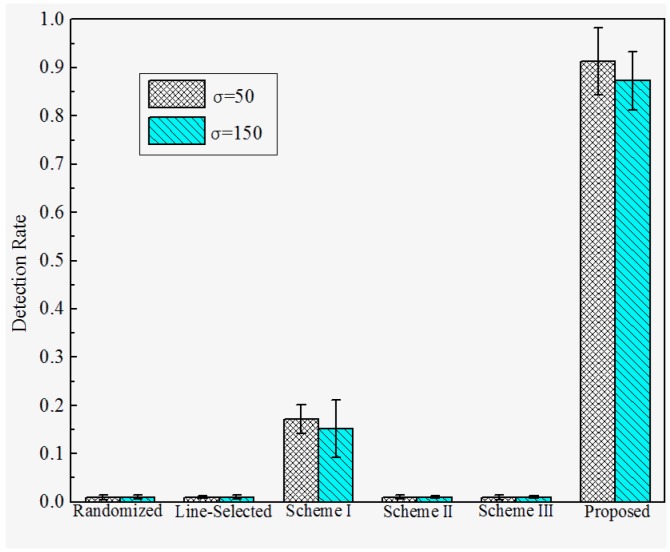
Detection rate of proposed versus prior works under attack strategy III when *D* = 200.

**Detection Rate under Attack Strategy III**. The detection rates under attack strategy III are shown in Figure 11 and Figure 12. We observe that the Randomized Multicast, Line-selected Multicast, and Scheme II and III have almost no resilience to attack strategy II. This is because all of these schemes do not take the case that replica nodes generate falsified location claims into consideration. Once the adversary generates the falsified location claim that is identical to their origin compromised nodes, the multicast-family schemes will accept the replicas as benign nodes since the falsified location claim will bypass the confliction detection whereas Schemes II and III will detect the location anomaly of the falsified location claim and send them to their home group, the confliction detection will still accept these falsified claims since they are exactly the same as their original compromised nodes. Nevertheless, Scheme I can detect the replicas, which limits the damage of attack strategy III to some extents. This is because the trusted neighbor detection will block the suspicious replicas’ communication. However, note that the detection of Scheme I will still be disabled when the adversary combines attack strategies I and III. In contrast, our proposed scheme reveals the significant advantage of resilience to attack strategy III since we use a neighboring-based location similarity estimation. For all of the network configurations, our scheme has at least 85% probability to detect the replicas which adopt attack strategy III. Furthermore, the detection rate becomes higher with the increase of distance *D*, as well as the decrease of the deployment standard deviation σ. The reason behind such a tendency is quite similar to the tendency of the detection rate under attack strategy II.

**Figure 13:**
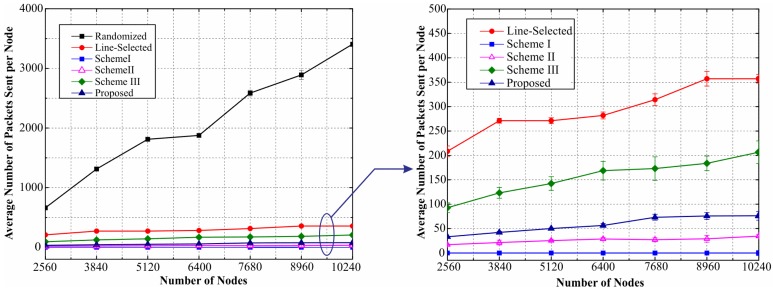
Communication overhead of the proposed and previous works.

**Comparison of Communication Overhead.** We investigate the communication overhead of the proposed scheme with various number of total nodes in the network, and compare it with the previous schemes. We set $P_{a}=0.3\;$ and $P_{s}=0.8$ while varying *M* from 2560 to 10,240. The corresponding simulation results are shown in Figure 13. As illustrated in Figure 13, the simulation results on the communication overhead are closely match our theoretical analysis in Section 6.1. The communication overhead of our scheme only grows at $O\left(\sqrt{M}\right)$ with the number of nodes *M* in the network. The value of communication overhead of our proposal falls in between that of Scheme II and Scheme III. When the number of nodes ranges from 2560 to 10,240, our scheme requires each node to transmit up to an average of 76.3628 packets. This is because, in our scheme, the nodes are required to reply the LAQ in addition to sending location claims. However it is worthwhile to point out that compared with the aforementioned Scheme II and Scheme III, in our scheme fewer sensor nodes are involved in the location claim transmission, which results in a lower value of total communication overhead for the entire network.

## 7. Conclusions

We have proposed a collaborative replica detection scheme for sensor networks that takes advantages of location similarity estimation techniques to provide high resiliency to a variety of dangerous attack strategies. Our scheme detects the replicas by verifying the authenticity and consistency of the nodes’ location claims using the neighborhood relationship derived from the group deployment knowledge. Specifically, we introduce the metric neighboring vector based location similarity (NV-LS) in this work to quantify the difference between the true and claimed location of each node in the network, and perform a threshold decision to find the replicas. Compared with the previous works, our scheme provides additional security services to prevent the replica nodes from falsifying their location claims without deploying resource-consuming localization algorithms on the resource-constraint sensor nodes.

Additionally we present heuristic analyses to evaluate the security strength against potentially effective attack strategies and show that the adversary’s benefits are significantly limited by the constraint of the proposed scheme for all the presented attack strategies. Furthermore, we evaluate the effectiveness and efficiency of our approach through theoretical analysis and simulation experiments, and compare them with that of prior works. Both of the results demonstrate that our approach provides better security resilience against the presented types of attack strategies in terms of a higher detection rate with a reasonable increase of communication overhead, as well as lower costs of computation and storage overhead.

## Figures and Tables

**Table 1:** Frequently-used notations in this paper.

$R_{z}$	the communication radius of sensor nodes and beacons	$k_{\langle i,\;j\rangle }$	secret key shared between node *i* and *j*
$d_{th}$	the trust threshold	$k_{\langle i,\;BS\rangle }$	secret key shared between node *i* and BS
$\tau_{CD}$	the threshold of confliction detection	$C_{k_{\mathrm{prv}}}$	the certification signed by BS
$\tau_{\mathrm{RD}}$	the threshold of replica detection	LAQ/LAR	the location authentication request/reply
$S_{nei-D}$	the derived neighboring vector	NAN	the node authentication needed request
$S_{ob}$	the observed neighboring vector	LCQ/LCD	the location claim request/decision
$k_{\mathrm{prv}}/k_{\mathrm{pub}}$	private/public key of BS		

**Table 2:** Communication overhead comparison.

Scheme	Communication Overhead
Randomized Multicast [6]	$O\left(k^{*}M^{2}\right)$
Line-selected Multicast [6]	$O\left(k^{*}\cdot M\sqrt{M}\right)$
Location Claim Scheme I [11]	Negligible
Location Claim Scheme II [11]	$O\left(P_{a}\cdot M\sqrt{M}\right)$
Location Claim Scheme III [11]	$O\left(P_{a}\cdot M\sqrt{M}\cdot \log_{2}\left(m\right)\right)$
Our proposed scheme	$O\left(P_{a}\cdot \left(M{\bar{N}}_{\mathrm{nei}}+M\sqrt{M}\right)\right)$

* *k* is the rounds of location claim executed in [1].

**Table 3:** Computation overhead comparison.

Scheme	Computation Overhead
Randomized Multicast [6]	$O\left(k^{*}\sqrt{M}\right)$
Line-selected Multicast [6]	$O\left(k^{*}\sqrt{M}\right)$
Location Claim Scheme I [11]	Negligible
Location Claim Scheme II [11]	$O\left({\bar{N}}_{nei}+P_{a}\cdot \left(\frac{M}{m}P_{s}^{**}+\sqrt{M}\right)\right)$
Location Claim Scheme III [11]	$O\left({\bar{N}}_{nei}+P_{a}\cdot \left(\frac{M}{m}P_{s}^{**}+\log_{2}\left(m\right)\sqrt{M}\right)\right)$
Our proposed scheme	$O\left({\bar{N}}_{\mathrm{nei}}\left(f_{c}+\left(1-f_{c}\right)P_{a}\right)+\left(f_{c}+\left(1-f_{c}\right)P_{a}\right)\right)$

^**^ Ps is the probability that the nodes in the replica’s home group caches the replica’s location claim.

**Table 4:** Storage overhead comparison.

Scheme	Claim Storage Overhead
Randomized Multicast [6]	$O\left(k^{*}\sqrt{M}\right)$
Line-selected Multicast [6]	$O\left(k^{*}\sqrt{M}\right)$
Location Claim Scheme I [11]	Negligible
Location Claim Scheme II [11]	$O\left({\bar{N}}_{nei}+P_{a}\cdot \frac{M}{m}\cdot p_{s}\right)$
Location Claim Scheme III [11]	$O\left({\bar{N}}_{nei}+P_{a}\cdot \frac{M}{m}\cdot \log_{2}\left(m\right)\cdot p_{s}\right)$
Our proposed scheme	Negligible

**Table 5:** Simulation parameters.

Parameter	Value
Power decay in reference distance (*A*)	55 dB
Maximum data rate	250 Kbps
Packet size	36 Bytes
Average radio noise floor	−110 dBm
Standard deviation for WGN	4.0 dB
Receiving Sensitivity	−105 dBm
